# Randomized Controlled Trial Examining the Effects of Fish Oil and Multivitamin Supplementation on the Incorporation of *n*-3 and *n*-6 Fatty Acids into Red Blood Cells

**DOI:** 10.3390/nu6051956

**Published:** 2014-05-14

**Authors:** Andrew Pipingas, Robyn Cockerell, Natalie Grima, Andrew Sinclair, Con Stough, Andrew Scholey, Stephen Myers, Kevin Croft, Avni Sali, Matthew P. Pase

**Affiliations:** 1 Centre for Human Psychopharmacology, Swinburne University of Technology, P.O. Box 218, Hawthorn 3122, Australia; E-Mails: robyn_cockerell@hotmail.com (R.C.); cstough@gmail.com (C.S.); ascholey@swin.edu.au (A.S.); mpase@swin.edu.au (M.P.P.); 2 Faculty of Medicine, Nursing and Health Sciences, Monash University, Clayton VIC 3800, Australia; E-Mail: nataliegrima@gmail.com; 3 Metabolic Research Unit, School of Medicine, Deakin University, Melbourne 3216, Australia; E-Mail: andrew.sinclair@deakin.edu.au; 4 NatMed-Research Unit, Southern Cross University, Lismore NSW 2480, Australia; E-Mail: stephen.myers@scu.edu.au; 5 School of Medicine and Pharmacology, University of Western Australia, Crawley 6009, Australia; E-Mail: kevin.croft@uwa.edu.au; 6 National Institute of Integrative Medicine, Hawthorn 3122, Australia; E-Mail: asali@niim.com.au

**Keywords:** fish oils, multivitamins, *n*-3, randomized, controlled trial (RCT), fatty acid, diet

## Abstract

The present randomized, placebo-controlled, double-blind, parallel-groups clinical trial examined the effects of fish oil and multivitamin supplementation on the incorporation of *n*-3 and *n*-6 fatty acids into red blood cells. Healthy adult humans (*n* = 160) were randomized to receive 6 g of fish oil, 6 g of fish oil plus a multivitamin, 3 g of fish oil plus a multivitamin or a placebo daily for 16 weeks. Treatment with 6 g of fish oil, with or without a daily multivitamin, led to higher eicosapentaenoic acid (EPA) composition at endpoint. Docosahexaenoic acid (DHA) composition was unchanged following treatment. The long chain LC *n*-3 PUFA index was only higher, compared to placebo, in the group receiving the combination of 6 g of fish oil and the multivitamin. Analysis by gender revealed that all treatments increased EPA incorporation in females while, in males, EPA was only significantly increased by the 6 g fish oil multivitamin combination. There was considerable individual variability in the red blood cell incorporation of EPA and DHA at endpoint. Gender contributed to a large proportion of this variability with females generally showing higher LC *n*-3 PUFA composition at endpoint. In conclusion, the incorporation of LC *n*-3 PUFA into red blood cells was influenced by dosage, the concurrent intake of vitamin/minerals and gender.

## 1. Introduction

Two of the most commonly consumed dietary supplements in the Western world are fish oils containing long chain *n*-3 polyunsaturated fatty acids (LC *n*-3 PUFA) and multivitamins [1,2]. The high prevalence of multivitamin use can be attributed to the fact that vitamin deficiencies are common, even in affluent countries [3]. The high prevalence of fish oil use may be in response to recent health messages, made by respected medical authorities such as the American Heart Association, advocating the benefits of increasing dietary LC *n*-3 PUFA intake.

Extensive research has explored the effects of multivitamin and fish oil supplementation in isolation, however, examination into their combined effect on human health remains scarce. Data from the National Health and Nutrition Examination Survey suggests that users of complementary medicine are most likely to use more than one supplement [4] meaning that many people are using both vitamin and fish oil supplements at the same time. There is also some preliminary evidence to suggest that vitamins and fish oils may have synergistic effects. Vitamin and mineral co-factors can influence the biosynthesis of LC *n*-3 PUFA, altering levels of LC *n*-3 PUFA measured *in vivo* [5,6,7]. In particular, a preclinical study demonstrated that an experimentally induced folic acid deficiency was associated with a fall in LC *n*-3 PUFA levels, suggesting that low levels of antioxidant vitamins may increase lipid peroxidation [7]. Based on their frequency of use and potentially synergistic actions, there is a clear need to understand how multivitamins and fish oils combine to affect potential health outcomes.

The present study investigated the effects of fish oil supplementation, with and without the addition of a multivitamin, on LC *n*-3 PUFA and LC *n*-6 PUFA incorporation measured in red blood cells. Healthy elderly participants (*n* = 160) were randomized into four groups to receive daily: (1) 6 g of fish oil; (2) 6 g of fish oil plus a multivitamin; (3) 3 g of fish oil and a multivitamin; or (4) a placebo in a double-blind, parallel groups design. The primary outcome of this trial was the effect of treatment on cognitive and cardiovascular function, which has been previously published [8]. This paper is concerned with the secondary aim of this trial which was to examine how high and low dosages of fish oil, in combination with a multivitamin, affected the incorporation of LC *n*-3 PUFA into erythrocytes. Specifically, in participants taking fish oil, we predicted increases in both EPA and DHA given that these were provided in balanced proportions in the fish oil supplements; and a dose response effect between consumption of 3 g and 6 g of fish oil. Additionally, we also examined whether combining 6 g of fish oil with a daily multivitamin increased LC *n*-3 PUFA red blood cell incorporation, over and above the effects of fish oil alone.

## 2. Methods

### 2.1. Participants

**Figure 1:**
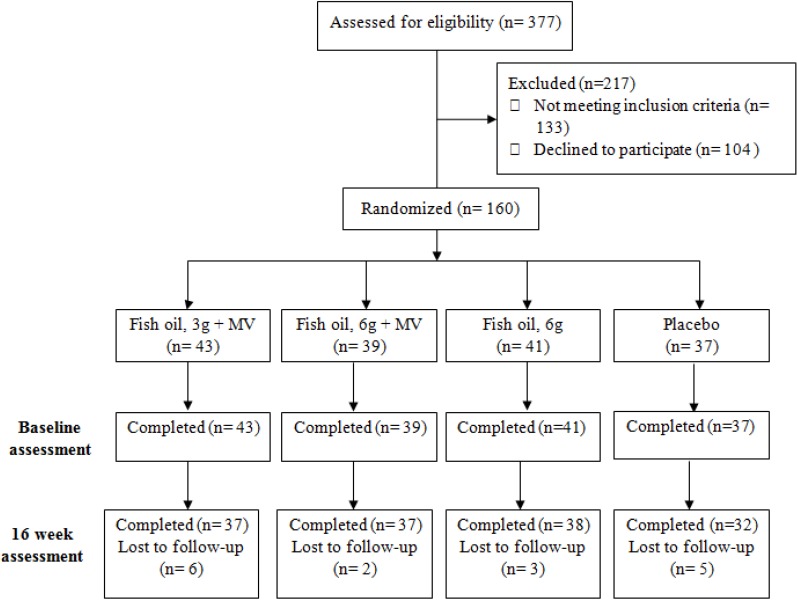
Participant flow diagram. MV: multivitamin.

The sample consisted of 160 healthy male and female volunteers aged 50 to 70 years. Participants were recruited from the general community and were non-smoking volunteers, not currently taking any medication or vitamin/herbal supplements. Exclusion criteria were; diagnosis of dementia, diabetes, neurologic (*i.e.*, Epilepsy, Parkinson’s disease, head trauma) or psychiatric disorders (*i.e.*, depression, schizophrenia), cardiovascular disease (including stroke) or past or present drug or alcohol abuse. Individuals taking anti-coagulant, anti-cholinergic, anti-depressants or acetyl-cholinesterase inhibitors were also excluded. Further exclusion criteria included those currently taking cognitive enhancing supplements regularly and current or long-term multivitamin or fish oil supplementation. The participant flow diagram is shown in Figure 1. The study randomized 160 participants and 144 completed the trial.

### 2.2. Setting

The study was conducted at Swinburne University of Technology, Hawthorn, Australia.

### 2.3. Interventions, Randomization and Blinding

The trial was randomised, placebo-controlled and double-blind, using a parallel group design. The participants were randomly assigned to one of the following four daily treatments:

(1) Multivitamin combined with 3 g of fish oil (240 mg EPA and 240 mg DHA);
(2) Multivitamin combined with 6 g of fish oil (480 mg EPA and 480 mg DHA);
(3) Placebo multivitamin combined with 6 g fish oil (480 mg EPA and 480 mg DHA);
(4) Placebo multivitamin combined with placebo fish oil (Sunola oil).

Participants consumed their assigned treatment daily for 16 weeks. The clinical trials supplements and matching placebos were provided by Swisse Wellness Pty Ltd. (Melbourne, Australia). The active fish oil supplement was Swisse Ultiboost Wild Salmon Oil and the active multivitamin supplement was Swisse Ultivite 50+ (Mens and Womens formulations). The constituents of the multivitamins are given in the online supplement (Table S1). All participants took one multivitamin (or its corresponding placebo) daily. Participants allocated to receive 6 g of fish oil daily were required to take six active fish oil capsules daily. Participants randomized to receive 3 g of fish oil took three active fish oil capsules and three matching placebo capsules daily. Participants in the placebo group received six placebo fish oil capsules daily. The placebo fish oil contained 1000 mg of Sunola Oil and 50 IU of vitamin E administered in a soft gelatin capsule. Sunola oil is a mono-unsaturated, high oleic (*n*-9) sunflower oil and was chosen as a control given that it is virtually *trans*-fat free and has a similar profile to olive oil. Small sachets with a few drops of fish oil were included in containers to assist with blinding by providing a fish odour when opened. The placebo multivitamin contained carrot powder with a small amount of riboflavin to produce colouration of the urine similar to the active multivitamin. The placebos were identical to the active tablets in shape, size and colour.

Participants were randomly assigned to one of the four experimental groups using a random permuted block procedure with a block size of four. The randomisation was conducted independently by the supplement supplier and the bottles labeled according to the randomization schedule. The research staff were blinded to this allocation. To ensure adequate blinding, placebo and active treatments were packaged in identical blister packs for multivitamins and sealed plastic containers for fish oil capsules. Participants were allocated the next sequential number upon enrolment in the study. Data was unblinded following the analysis of the main study aims.

### 2.4. Outcomes Measures

The outcome measures for this study were the incorporation of LC *n*-3 PUFA and LC *n*-6 PUFA fatty acids into red blood cells, following supplementation. Blood sampling was conducted in the morning following a 12-h fasting period. Blood was collected via venepuncture from the antecubital vein, using the BD-vacutainer system. Samples were analysed by Healthscope Functional Pathology according to standard procedures. Samples were centrifuged at 3000 rpm for 10 min before plasma was removed. Red blood cells were then washed twice by suspending in 0.9% saline, centrifuging at 3000 rpm aspirating off the supernatant. Red cells were then stored at −20 °C until assayed. Methyl ethers of fatty acids were prepared as follows: 350 μL of plasma and 1.5 mL of red cell extract were added to a 10 mL extraction tube. 3.8 mL of a methanol/chloroform mixture was added before vortexing the tube for 6 min. 0.8 mL of 0.1 M KCI solution was added and the tube was then vortexed for a further 3 min and then centrifuged at 3000 rpm for 10 min. The upper aqueous layer was discarded by aspiration. A silane treated glass wool was placed in the bottom of a glass Pasteur pipette and then filled with sodium sulphate. The organic layer was passed through sodium sulphate and the eluate was collected in 2 mL vials. The solvent was evaporated to dryness in heating block (<45 °C) with nitrogen. The dry residue was reconstituted with 130 μL of Meth-Prep II methylation agent. Vials were then closed and left to rest at room temperature overnight. 0.8 μL of the esterification mixture was then injected into a Gas Liquid Chromatography using flame ionisation detection (GLC-FID), on a Schimadzu G-2010 (Shumadzu, Kyoto, Japan), for analysis. Chromatic conditions included a detector temperature of 300 °C and injector temperature of 250 °C. Injector sampling time was 0.5 min. All fatty acid values were expressed as a percentage of red blood cell total fatty acids. The LC *n*-3 PUFA index was calculated as total DHA + total EPA + total DPA. Total *n*-3 fatty acid and total *n*-6 fatty acids were calculated as the combination of both total long and short chain *n*-3 (alpha linolenic acid + EPA + DHA + DPA) and *n*-6 (linoleic acid + gamma linolenic acid + eicosadienoic acid + eicosatrienoic acid + arachidonic acid) fatty acids respectively.

### 2.5. Sample Size

The sample size of 160 was determined based on the variance of the cognitive and cardiovascular study outcomes, which are reported separately [8]. Percept changes in cognitive performance were expected to be considerably smaller than changes in LC *n*-3 PUFA over the study period. Thus, the study was believed to be appropriately powered to investigate changes in LC *n*-3 PUFA due to treatment.

### 2.6. Procedure

Participants were required to attend testing sessions at our laboratories on three separate occasions; at baseline, following six weeks of supplementation and following 16 weeks of supplementation. Blood samples were taken both at baseline and at week 16 only.

The research was conducted in accordance with the guidelines of the Australian National Health and Medical Research Council and the Declaration of Helsinki (as revised in 2004). The study was approved by the Swinburne University Human Research Ethics Committee. Written informed consent was obtained from all subjects. This trial was registered with the Australian and New Zealand Clinical Trial Registry (ACTRN12611000094976).

### 2.7. Statistical Analyses

Results were analyzed using SPSS statistics (IBM, version 20, New York, NY, USA). Univariate analyses of variance (ANOVA) were used to examine whether any significant group differences existed at baseline for the basic demographic and health variables displayed in Table 1. Univariate ANCOVAs were also used to examine the effects of treatment on all outcomes variables at week 16. Significant main effects of treatment were further examined using simple planned contrasts, applying Bonferroni corrections to each contrast in order to adjust for comparisons across the treatment groups. Given that males and females may respond differently to LC *n*-3 PUFA supplementation across different clinical outcomes, we examined whether gender predicted the incorporation of LC *n*-3 PUFA and LC *n*-6 PUFA into red blood cells. For these analyses, gender was entered as a fixed factor and the respective fatty acid variable at endpoint as the dependent variable in ANOVA. All analyses were adjusted for the respective scores at baseline and all results were considered statistically significant at *p* < 0.05.

## 3. Results

The trial started in 2010 and was ceased in 2012 due to attainment of the desired sample size. No serious adverse events were reported.

### 3.1. Cohort Demographics

The descriptive demographics of the study population at baseline are given in Table 1. The mean age of the sample was 59 years. The sample was roughly gender balanced with slightly more females. On average, the sample was well educated and high functioning. Blood pressure levels were normal across the sample although Low Density Lipoprotein (LDL) cholesterol tended to be elevated across all treatment groups. ANOVA revealed that, at baseline, the treatment groups were well matched across all continuous variables displayed in Table 1, with no significant group differences noted. ANOVA also suggested that males and females were well matched across all fatty acid variables at baseline. Mean baseline fatty acid values can be seen in Table 2, stratified according to treatment allocation. Across the whole sample, median red blood cell composition of *n*-3 fatty acid and *n*-6 fatty acid tended to be lower than those reported in a normative group of almost 160,000 people [9]. In contrast, median baseline values of saturated and monounsaturated fats were higher in the present cohort.

**Table 1:** Sample demographics (means and standard deviations) stratified by treatment allocation.

Variable	Fish Oil, 3 g + Multivitamin	Fish Oil, 6 g + Multivitamin	Fish Oil, 6 g	Placebo	Overall
*N*	43	39	41	37	160
Age, year	59.48 (5.64)	58.90 (5.60)	59.51 (5.89)	59.19 (5.96)	59.28 (5.72)
Male, %	48	48	46	46	47
Education, year	15.54 (3.10)	15.79 (3.92)	15.84 (3.94)	15.76 (3.38)	15.73 (3.57)
MMSE	28.12 (2.04)	28.25 (1.61)	28.07 (2.02)	28.14 (1.74)	28.14 (1.85)
Height, cm	170.79 (8.79)	169.63 (9.17)	173.03 (9.91)	170.23 (9.05)	170.93 (9.24)
Weight, kg	74.88 (13.78)	70.98 (12.08)	76.35 (16.28)	70.35 (11.01)	73.18 (13.57)
BMI	25.54(3.59)	24.41 (3.07)	25.31 (4.03)	24.2 (2.79)	24.88 (3.43)
LDL, mmol/L	3.31 (0.71)	3.51 (0.72)	3.37 (0.84)	3.27 (0.72)	3.36 (0.75)
HDL, mmol/L	1.52 (0.42)	1.61 (0.39)	1.56 (0.44)	1.57 (0.36)	1.56 (0.40)
SBP, mmHg	125.81 (20.97)	122.59 (16.92)	126.29 (16.92)	121.41 (21.47)	124.12 (19.10)
DBP, mmHg	77.19 (13.32)	75.46 (10.90)	77.71 (9.82)	74.62 (12.13)	76.30 (11.58)

Note: MMSE: Mini Mental State Examination, BMI: Body Mass Index, LDL: low density lipoprotein cholesterol, HDL: high density lipoprotein cholesterol, SBP: systolic blood pressure, diastolic blood pressure.

### 3.2. Main Effects of Treatment on n-3 Fatty Acid and n-6 Fatty Acid Blood Measures

Table 2 displays red blood cell fatty acid composition before and after treatment. Univariate ANCOVA revealed that week 16 EPA (*F*(3, 136) = 12.20, *p* < 0.001), DPA (*F*(3, 136) = 3.09, *p* < 0.05), LC *n*-3 PUFA index (*F*(3, 136) = 3.98, *p* < 0.01), AA/EPA ratio (*F*(3, 136) = 53.74, *p* < 0.001), total *n*-3 fatty acid (*F*(3, 136) = 3.96, *p* < 0.05), total *n*-6 (*F*(3, 136) = 4.01, *p* < 0.01) and the *n*-3/*n*-6 ratio (*F*(3, 136) = 10.13, *p* < 0.001) differed between treatment groups, when controlling for baseline. Week 16 DHA (*F*(3, 136) = 2.01, *p* = 0.10) did not differ according to treatment allocation, when controlling for baseline.

**Table 2:** Means, standard deviations and percentage change for red blood cell fatty acid status over the course of supplementation.

Variable	Fish Oil,	Fish Oil,	Fish Oil, 6 g	Placebo	ANCOVA
	3 g + Multivitamin	6 g + Multivitamin			*F* Value
EPA, %					12.20 ***
Baseline	0.99 (0.46)	1.01 (0.30)	1.06 (0.41)	1.00 (0.43)	
Week 16	1.41 (0.68)	1.98 (0.65) ***	1.66 (0.75) ***	1.06 (0.48)	
% change	42.42	96.04	56.60	6.00	
DHA, %					2.01
Baseline	2.66 (1.26)	2.74 (0.96)	2.92 (1.10)	2.82 (1.11)	
Week 16	2.94 (1.58)	3.64 (1.27)	3.16 (1.60)	2.86 (1.21)	
% change	10.53	32.85	8.22	1.42	
DPA, %					3.09 *
Baseline	1.69 (0.75)	1.88 (0.50)	1.94 (0.65)	1.83 (0.61)	
Week 16	1.74 (0.85)	2.31 (0.67)	1.93 (0.96)	1.87 (0.74)	
%change	2.96	22.87	−0.52	2.19	
LC *n*-3 PUFA index, %					3.98 **
Baseline	5.34 (2.31)	5.63 (1.59)	5.92 (2.04)	5.65 (2.00)	
Week 16	6.11 (2.99)	7.92 (2.46) **	6.75 (3.23)	5.79 (2.31)	
% change	14.42	40.67	14.02	2.48	
AA/EPA, ratio					53.74 ***
Baseline	10.18 (4.06)	10.18 (2.24)	9.75 (3.20)	10.58 (3.79)	
Week 16	6.11 (2.22) ***	4.48 (1.05) ***	4.64 (1.18) ***	9.72 (3.13)	
% change	−39.98	−55.99	−52.41	−8.13	
Total *n*-3 FA, %					3.96 *
Baseline	5.52 (2.32)	5.83 (1.61)	6.11 (2.06)	5.85 (2.01)	
Week 16	6.26 (3.00)	8.10 (2.47) **	6.90 (3.22)	5.99 (2.32)	
% change	13.41	38.94	12.93	2.39	
Total *n*-6 FA, %					4.01 **
Baseline	21.97 (4.82)	24.27 (3.42)	23.80 (4.50)	23.41 (4.49)	
Week 16	19.77 (5.60)*	21.63 (4.34)	19.98 (5.53) *	23.66 (4.54)	
% change	−10.01	−10.88	−16.05	1.07	
*n*-3/*n*-6 FA, ratio					10.13 ***
Baseline	0.24 (0.78)	0.23 (0.06)	0.25 (0.07)	0.24 (0.06)	
Week 16	0.29 (0.10)	0.37 (0.09) ***	0.33 (0.11) **	0.25 (0.08)	
% change	20.83	60.86	32.00	4.17	
*n*	35	37	38	31	

Note: EPA: Eicosapentaenoic Acid, DHA: Docosahexaenoic Acid, AA: Arachidonic Acid, LC *n*-3 PUFA: Long chain *n*-3 polyunsaturated fatty acid, FA: Fatty acid, ANOVA results are for univariate analysis of variance comparing blood values at end-point, by treatment allocation, whilst controlling for baseline values. Stars display results of simple planned contrasts between the placebo and treatment groups. * *p* < 0.05, ** *p* < 0.01, *** *p* < 0.001.

### 3.3. Effects of Treatment, Relative to Placebo, on n-3 Fatty Acid and n-6 Fatty Acid Blood Measures

Those treatment groups differing significantly from placebo can be seen in Table 2. The week 16 AA/EPA ratio was lower across all treatment groups relative to placebo. EPA was significantly higher at week 16 in the two 6 g fish oil groups. Despite a significant main effect, none of the treatment groups had significantly higher DPA at study endpoint, as compared to placebo. Both the LC *n*-3 PUFA index and total *n*-3 fatty acid were only higher in the group receiving the combination of the 6 g fish oil and multivitamin combination. *n*-6 Fatty acid was lower in the 3 g fish oil multivitamin group as well as in the group receiving 6 g of fish oil in isolation. The *n*-3/*n*-6 fatty acid ratio was significantly higher following 6 g of fish oil supplementation, irrespective of the multivitamin.

### 3.4. Effects of Fish Oil Dosage on n-3 Fatty Acid and n-6 Fatty Acid Blood Measures

Red blood cell incorporation of *n*-3 fatty acid at week 16 was expected to be higher following supplementation with 6 g as opposed to 3 g of fish oil, demonstrating a dose response. When directly comparing the 6 g fish oil multivitamin group to the 3 g fish oil multivitamin group, the higher dose fish oil group displayed significantly higher week 16 incorporation of EPA (*p* < 0.01), DPA (*p* < 0.05), LC *n*-3 PUFA index (*p* < 0.05), total *n*-3 fatty acid (*p* < 0.05) and the *n*-3/*n*-6 fatty acid ratio (*p* < 0.01). The AA/EPA ratio was also lower in the higher dose fish oil group (*p* < 0.01).

### 3.5. Effects of Combining Fish Oil with a Multivitamin on n-3 and n-6 Blood Measures

Adding a multivitamin to the fish oil was expected to increase week 16 *n*-3 fatty acid incorporation into red blood cells, over and above the effects of fish oil alone. When directly comparing the two 6 g fish oils groups, with and without the addition of a multivitamin, there were no significant differences between the two groups across any of the week 16 *n*-3 fatty acid or *n*-6 fatty acid variables. However, when comparing to placebo, the LC *n*-3 PUFA index and total *n*-3 fatty were only increased following the 6 g fish oil multivitamin combination (Table 2) and not the 6g fish oil alone.

### 3.6. Sources of Variability in Red Blood Cell n-3 Fatty Acid Incorporation

**Figure 2:**
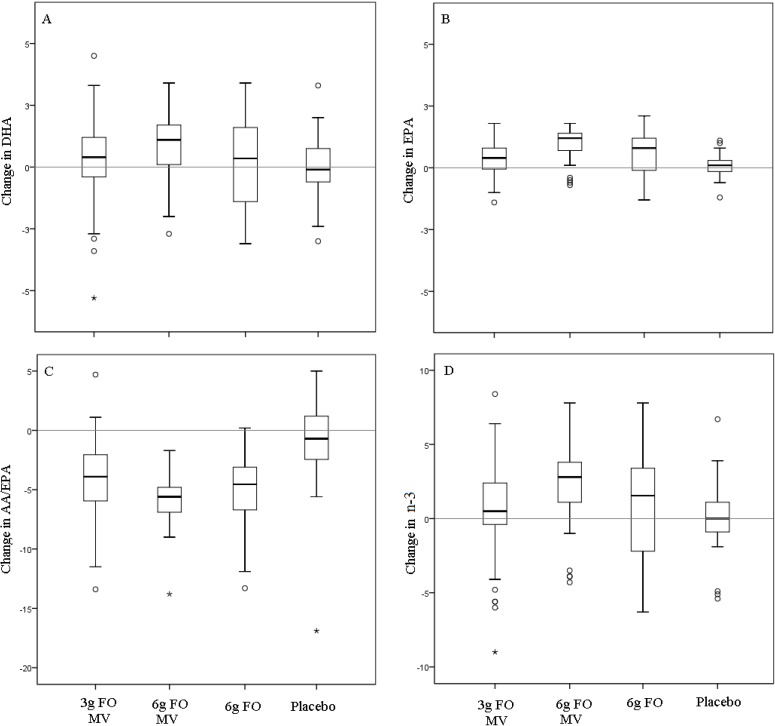
Variability in red blood cell incorporation stratified by treatment allocation for measures of DHA (**A**), EPA (**B**), AA/EPA ratio (**C**) and total *n*-3 fatty acid (**D**). EP: eicosapentaenoic acid, DHA:docosahexaenoic acid, AA: Arachidonic acid, FO: fish oil, MV: multivitamin. Circles and stars represent outliers less than 2 and greater than 2 standard deviations from the mean respectively.

Figure 2 shows changes in total *n*-3 fatty acid, EPA, DHA and the AA/EPA ratio, over the course of the study, stratified by treatment allocation. Considerable individual variability in *n*-3 fatty acid change is evident. Interestingly, many participants allocated to the fish oil conditions decreased their amount of total *n*-3 fatty acid and DHA as measured from red blood cells over the 16 week study period. However, EPA tended to increase and the AA/EPA ratio decreased in the fish oil treatment arms suggesting that the variability in DHA and total *n*-3 fatty acid may reflect individual differences in incorporation rather than compliance to treatment. The AA/EPA ratio appears to be the best indicator of compliance to treatment as almost all participants receiving active fish oil decreased their ratio, whilst those in the control group tended to remain stable. Across all *n*-3 fatty acid measures, the coefficients of variations using week 16 treatment means and standard deviations (Table 3) tended to be lowest for the 6 g fish oil + multivitamin group and highest for the 3 g fish oil + multivitamin group. The coefficients of variation also tended to be lowest for the AA/EPA ratio.

### 3.7. Gender and Variability in n-3 Fatty Acid

**Figure 3:**
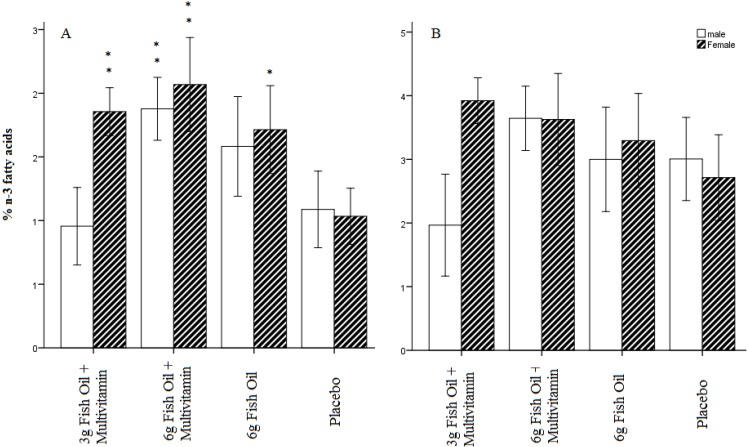
Incorporation of EPA (**A**) and DHA (**B**) into red blood cells at study endpoint stratified by treatment allocation and gender. Males are represented by white bars and females by shaded bars. * Group significantly different to placebo at *p* < 0.05, ** Group significantly different to placebo at *p* < 0.01.

Recent studies suggest that males and females respond differently to LC *n*-3 PUFA supplementation across different clinical outcomes [10,11]. Males and females may therefore differ in their ability to incorporate *n*-3 fatty acids into erythrocytes. The authors investigated whether gender accounted for some of the observed variability in the incorporation of *n*-3 fatty acids into red blood cells. Gender was a significant predictor of week 16 EPA (*F*(3, 135) = 4.54, *p* < 0.05), DHA (*F*(3, 135) = 4.42, *p* < 0.05), LC *n*-3 PUFA index (*F*(3, 135) = 4.53, *p* < 0.05) and total *n*-3 fatty acids (*F*(3, 135) = 5.10, *p* < 0.05). Gender was not predictive of the week 16 AA/EPA ratio (*F*(3, 135) = 0.01, *p* = 0.94) nor DPA (*F*(3, 135) = 2.58, *p* = 0.11). Significant interactions were also found between treatment allocation and gender for EPA (*F*(3, 135) = 3.40, *p* < 0.05), DHA (*F*(3, 135) = 4.99, *p* < 0.01), DPA (*F*(3, 135) = 5.10, *p* < 0.01), LC *n*-3 PUFA index (*F*(3, 135) = 5.37, *p* < 0.01) and total *n*-3 fatty acids (*F*(3, 135) = 4.86, *p* < 0.01). Selected interactions are displayed in Figure 3 (Separate analysis of males and females across all blood measures can be seen in Tables S2 and S3 of the online supplement). It can be seen that females tended to have higher red blood cell incorporation of most *n*-3 fatty acid blood measures at endpoint. The most interesting finding was that, in females, all treatment groups led to an increase in EPA relative to placebo (Figure 3A). In males, EPA only increased following treatment with the combination of 6 g fish oil and a daily multivitamin. Unlike gender, other demographic and clinical factors such as age, height, weight, physical activity, total cholesterol, high sensitivity CRP and general health status did not predict *n*-3 fatty acid incorporation into red blood cells at study endpoint.

**Table 3:** Coefficients of variation for each *n*-3 fatty acid blood measure at week 16, stratified by treatment allocation.

Variable	Fish Oil,	Fish Oil,	Fish Oil,	Placebo
	3g + Multivitamin	6g + Multivitamin	6 g	
EPA	0.48	0.33	0.45	0.45
DHA	0.54	0.35	0.51	0.42
DPA	0.49	0.29	0.50	0.40
LC *n*-3 PUFA index	0.49	0.31	0.48	0.40
AA/EPA	0.36	0.23	0.25	0.34
Total *n*-3	0.48	0.30	0.47	0.39
*n*-3/*n*-6	0.34	0.24	0.33	0.32

Note: EPA = Eicosapentaenoic Acid, DHA = Docosahexaenoic Acid, AA = Arachidonic Acid, LC *n*-3 PUFA = Long chain *n*-3 polyunsaturated fatty acid. Coefficients of variation calculated as week 16 standard deviation/mean for each measure respectively.

## 4. Discussion

This study investigated the effects of fish oil supplementation, with and without the addition of a multivitamin, on red blood cell fatty acid composition. Daily supplementation with 6 g of fish oil for 16 weeks led to higher composition of EPA as well as a lower AA/EPA ratio. The LC *n*-3 PUFA index and total *n*-3 fatty acid only increased after 6 g of fish oil was administered in combination with a daily multivitamin. As expected, 6 g of fish oil combined with a multivitamin was more effective at increasing *n*-3 fatty acid erythrocyte composition than 3 g of fish oil combined with a multivitamin. Over the 16 week study period there was considerable individual variability in *n*-3 fatty acid change, much of which was accounted for by gender. A predicted dose response effect in *n*-3 fatty acids was seen between the 3 g and 6 g fish oil supplements.

Fish oils combined with the concomitant use of a multivitamin increased the LC *n*-3 PUFA index. The LC *n*-3 PUFA index was not increased following 6 g of fish oil alone. It thus follows that the intake of vitamins and minerals, from dietary sources, may also affect the efficacy of fish oil supplementation. These results are important because low amounts of LC *n*-3 PUFA are associated with an increased risk of death from coronary heart disease [12]. Combining fish oil supplementation with adequate vitamin/mineral intake, either through diet or supplementation, may help bolster the LC *n*-3 PUFA index thus reducing cardiovascular disease risk. Future fish oil intervention trials are advised to account for habitual intake of vitamins (*i.e.*, through food frequency questionnaires), as this may partly explain individual differences in response to fish oil treatment.

The mechanism by which vitamin/mineral intake interacts with fish oil supplementation to increase the LC *n*-3 PUFA index is not completely understood. Preliminary evidence obtained from animal studies suggests that certain vitamins and minerals, such as B vitamins and iron, influence *in vivo* composition of *n*-3 [5,6,7]. Although speculative, multivitamin use may boost antioxidant defence, protecting LC *n*-3 PUFA from oxidation.

The present results suggest that considerable variability exists in the individual to uptake and transfer LC *n*-3 PUFA to red blood cells. Gender was identified as one factor contributing to this variability. Females supplemented with fish oil were generally found to have higher incorporation of total *n*-3 fatty acids at the end of the study. No gender differences were found for the AA/EPA ratio suggesting that gender differences are not merely due to compliance to treatment. Instead, these results suggest that males and females differ in their ability to incorporate some specific types of LC *n*-3 PUFA, such as EPA, into red blood cells. In females, all treatments led to a significant increase in EPA over and above the placebo. In contrast, only the 6 g fish oil multivitamin treatment led to an increase in EPA composition in males. These findings are interesting in light of recent studies showing that males and females respond differently to LC *n*-3 PUFA supplementation across clinical outcomes such as platelet aggregation [10] and cognitive performance [11]. If the present findings can be replicated, they may have significant implications for health policy and guidelines because males and females may be required to consume different amounts of fatty fish or fish oil supplements in order to achieve optimal LC *n*-3 PUFA blood composition.

In certain areas of investigation, inconsistencies have been reported regarding the health benefits of fish oil supplementation. For example, randomized controlled trials have produced conflicting results as to whether fish oil supplementation can enhance cognitive performance or mitigate cognitive decline in adults [11,13,14]. These conflicting results are surprising given that observational studies have been far more consistent in suggesting that *n*-3 fatty acid blood composition is associated with cognitive outcomes [15,16,17,18]. In light of the present findings, inconsistencies reported in fish oil intervention studies may be partly due to individual differences in the ability to incorporate LC *n*-3 PUFAs into cell membranes. These individual differences may be due to gender or vitamin/mineral intake, either through background diet or concomitant supplement use. Others have also suggested that genetic markers, such as the presence of the APOE e4 allele, may also affect response to fish oil supplementation [19]. To counteract the variability in response to fish oil supplementation, these results highlight the importance of including blood measures of LC *n*-3 PUFA status in future fish oil intervention studies.

The fish oil supplements in the current study contained a balanced ratio of EPA and DHA. Although EPA increased by as much as 96%, DHA red blood cell levels did not significantly increase following fish oil supplementation. These results are consistent with a previous report showing that EPA, as compared to DHA, was better incorporated into erythrocyte membranes following supplementation [20]. In this previous study, EPA increased by 300% while DHA only increased by 42% following 8 weeks of daily supplementation with 1296 mg EPA and 864 mg DHA. Previous studies have also shown that the uptake of DHA into erythrocyte membranes is more variable than that of EPA [20,21].

Limitations of the current study include the relatively small sample size, the relatively short follow-up period and the fact that LC *n*-3 PUFA composition was only measured at baseline and then again following 16 weeks of supplementation. Assessing red blood cell fatty acid incorporation at multiple time points, spread out over the intervention period, would provide a better indicator of *n*-3 fatty acid change across time. The multivitamin formulations used in the present study differed slightly for males and females and this may have inflated some of the observed gender differences. Furthermore, we did not monitor or examine how changes in other dietary factors may have influenced the reported results over the 16 week study period. Lastly, recent studies have shown health benefits of fish oil associated with higher dosages than that used in the present study [11,22] and it is possible that higher dosages would differentially affect *n*-3 fatty blood biomarkers.

## 5. Conclusions

Daily supplementation for 16 weeks with 6g of fish oil, with or without a multivitamin, led to higher EPA incorporation into erythrocytes. A dose response effect was demonstrated between 3 g and 6 g of fish oil on *n*-3 fatty acids. Treatment had no effect on DHA composition. At study endpoint, the LC *n*-3 PUFA index was only higher for those receiving a multivitamin in addition to 6 g of daily fish oil, suggesting that some vitamins/minerals aid the incorporation of LC *n*-3 PUFA into red blood cells. There was considerable individual variability in the response to supplementation with females, generally found to incorporate LC *n*-3 PUFA into red blood cells more effectively than males. Relative to placebo, all treatments increased EPA in females whereas only the 6 g fish oil multivitamin combination treatment increased EPA in males. These results suggest that some males may incorporate relatively low amounts of LC *n*-3 PUFA into red blood cells despite adhering to LC *n*-3 PUFA intake guidelines. This is an important area for future research because dietary recommendations around LC *n*-3 PUFA intake may need to be gender specific.

## Supplementary Files

Supplementary File 1 Supplementary Information (DOCX, 37 KB)
