# Supplementary material for: Randomized Controlled Trial Examining the Effects of Fish Oil and Multivitamin Supplementation on the Incorporation of n-3 and n-6 Fatty Acids into Red Blood Cells
Source: Nutrients. 2014 May 14;6(5):1956–70. doi: 10.3390/nu6051956 (PMC4042109; doi:10.3390/nu6051956)
Supplement: Supplementary File 1 — Supplementary Information (DOCX, 37 KB) [file nutrients-06-01956-s001.docx]

**Supplementary Information**

**Table S1.** Constituents of the study multivitamins (Swisse Men’s and Women’s Ultivite 50+ Years).

| **Vitamin** | **Men’s Multivitamin** | **Women’s Multivitamin** |
| --- | --- | --- |
| Vitamin A | 2500 IU | 2500 IU |
| Vitamin E | 30.25 IU | 24.2 IU |
| Vitamin B1 | 35 mg | 30 mg |
| Vitamin B2 | 35 mg | 30 mg |
| Vitamin B3 | 25 mg | 20 mg |
| Vitamin B5 | 75 mg | 64.13 mg |
| Vitamin B6 | 26 mg | 30 mg |
| Vitamin B12 | 120 mcg | 115 mcg |
| Vitamin D3 | 200 IU | 200 IU |
| Vitamin H | 200 mcg | 150 mcg |
| Folic Acid | 500 mcg | 500 mcg |
| Vitamin K | 70 mcg | 60 mcg |
| Vitamin C | 165.5 mg | 185.3 mg |
| Citrus Bioflavonoids Extract | 20 mg | 20 mg |
| Calcium Ortate | 100 mg (equiv. 10 mg Calcium) | 100 mg (equiv. 10 mg Calcium) |
| Magnesium Aspartale Dihydrate | 100 mg (equiv. 7.5mg Magnesium) | 100 mg (equiv. 7.5mg Magnesium) |
| Selenium | 65 mcg (equiv. 25 mcg Selenium) | 85 mcg (equiv. 25mcg Selenium) |
| Molybdenum Trioxide | 67.5 mcg | 67.5 mcg |
| Chromium | - | 50 mcg (equiv. 402 mcg  Chromium picolnate) |
| Manganese Amino Acid Chelate | 40 mg (equiv. 4 mg Maganese) | 30 mg (equiv. 3 mg Maganese) |
| Ferrous Fumarate | 16.01 mg (equiv. 5 mg Iron) | 16.01 mg (equiv. 5 mg Iron) |
| Copper Giusonate | 12.1 mg (equiv. 1.2 mg Copper) | 8.57 mg (equiv. 1.2 mg Copper) |
| Potassium Iodide | 196 mcg | 196 mcg |
| Zinc Amino Acid Chelate | 100 mg | 75 mg |
| Co-Enzyme Q10 | 3 mg | 2 mg |
| Lactobacillus Phamnosus | 80 million organisms | 80 million organisms |
| Lactobacillus Acidophilus | 80 million organisms | 80 million organisms |
| Bifidobacterium Longum | 35 million organisms | 35 million organisms |
| Cranberry | 1000 mg | 800 mg |
| St. Mary’s Thistle | 1700 mg | 1500 mg |
| Maidenhaire Tree | 1000 mg | 1000 mg |
| Damiana | - | 500 mg |
| Tribulus | 1000 mg | - |
| Skullcap | 50 mg | 50 mg |
| Grape Seed | 1000 mg | 1000 mg |
| Stinging Nettle | 100 mg | 100 mg |
| Globe Artichoke | 50 mg | 50 mg |
| Muirapuama | 200 mg | - |

**Table S1**. *Cont.*

| Aztec Marigold | 100 mg | 100 mg |
| --- | --- | --- |
| Turmeric | - | 100 mg |
| Ashwagandha | - | 500 mg |
| Saw Palmetto | 300 mg | - |
| Hawthorn | 100 mg | 100 mg |
| Silica Collodial Anhydrous | - | 20 mg |
| Brahmi | - | 50 mg |
| Lecithin Powder | 10 mg | 10 mg |
| Spearmint Oil | 2 mg | 2 mg |
| Fresh Bilberry | - | 100 mg |

equiv.: equivalent.

**Table S2.** Red blood cell fatty acid composition over the course of supplementation for males only.

| **Variable** | **Fish Oil, 3 g + Multivitamin  (*n* = 18)** | **Fish Oil, 6 g + Multivitamin  (*n* = 18)** | **Fish Oil, 6 g  (*n* = 17)** | **Placebo  (*n* = 16)** | **ANCOVA *F* Value** |
| --- | --- | --- | --- | --- | --- |
| EPA, % |  |  |  |  | 9.09 *** |
| Baseline | 0.89 (0.38) | 0.91 (0.35) | 1.16 (0.41) | 0.88 (0.41) |  |
| Week 16 | 0.96 (0.61) | 1.88 (0.50) ** | 1.58 (0.76) | 1.09 (0.56) |  |
| % change | 7.87 | 106.59 | 36.21 | 23.86 |  |
| DHA, % |  |  |  |  | 5.26 ** |
| Baseline | 2.58 (1.34) | 2.57 (1.14) | 3.17 (1.08) | 2.58 (1.23) |  |
| Week 16 | 2.00 (1.61) | 3.64 (1.01) | 3.00 (1.60) | 3.01 (1.23) |  |
| % change | −22.48 | 41.63 | −5.36 | 16.67 |  |
| DPA, % |  |  |  |  | 6.32 ** |
| Baseline | 1.67 (0.70) | 1.81 (0.61) | 2.08 (0.62) | 1.69 (0.73) |  |
| Week 16 | 1.23 (0.90) * | 2.34 (0.57) | 1.84 (0.90) | 2.01 (0.78) |  |
| % change | −26.35 | 29.28 | −11.54 | 18.93 |  |
| LCN3 index, % |  |  |  |  | 6.27 ** |
| Baseline | 5.14 (2.21) | 5.29 (1.96) | 6.42 (1.95) | 5.15 (2.27) |  |
| Week 16 | 4.15 (3.03) | 7.86 (1.89) | 6.42 (3.20) | 6.10 (2.42) |  |
| % change | −19.26 | 48.58 | 0.00 | 18.45 |  |
| AA/EPA, ratio |  |  |  |  | 18.50 *** |
| Baseline | 11.05 (4.68) | 10.69 (2.40) | 8.76 (2.67) | 11.08 (4.42) |  |
| Week 16 | 6.51 (2.84) *** | 4.73 (1.01) *** | 4.76 (1.00) *** | 9.95 (3.46) |  |
| % change | −41.09 | −55.19 | −45.66 | −10.20 |  |
| Total *n*-3, % |  |  |  |  | 6.32 ** |
| Baseline | 5.32 (2.23) | 5.48 (1.99) | 6.60 (1.97) | 5.34 (2.29) |  |
| Week 16 | 4.29 (3.05) | 8.04 (1.92) | 6.55 (3.21) | 6.29 (2.44) |  |
| % change | −19.36 | 46.72 | −0.76 | 17.79 |  |
| Total *n*-6, % |  |  |  |  | 5.91 ** |

**Table S2**. *Cont.*

| Baseline | 22.28 (5.20) | 23.76 (4.78) | 24.00 (3.99) | 21.95 (5.90) |  |
| --- | --- | --- | --- | --- | --- |
| Week 16 | 16.96 (6.76) ** | 22.37 (3.65) | 19.44 (5.81) | 23.29 (4.37) |  |
| % change | −23.88 | −5.85 | −19.00 | 6.10 |  |
| *n*-3/*n*-6, ratio |  |  |  |  | 8.19 *** |
| Baseline | 0.23 (0.07) | 0.22 (0.06) | 0.27 (0.06) | 0.23 (0.07) |  |
| Week 16 | 0.22 (0.10) | 0.36 (0.07)* | 0.32 (0.10) | 0.26 (0.09) |  |
| % change | -4.35 | 63.64 | 18.52 | 13.04 |  |

Note: EPA = [Eicosapentaenoic](http://en.wikipedia.org/wiki/Eicosapentaenoic_acid) Acid, DHA = [Docosahexaenoic Acid](http://en.wikipedia.org/wiki/Docosahexaenoic_acid), LCN3 = Omega-3, AA = Arachidonic Acid. ANOVA results are for univariate analysis of variance comparing blood values at end-point, by treatment allocation, whilst controlling for baseline values. Stars display results of simple planned contrasts using the placebo as the reference group. * *p* < 0.05, ** *p* < 0.01, *** *p* < 0.001.

**Table S3.** Red blood cell fatty acid composition over the course of supplementation for females only.

| **Variable** | **Fish Oil, 3 g + Multivitamin  (*n* = 17)** | **Fish Oil, 6 g + Multivitamin  (*n* = 19)** | **Fish Oil, 6 g  (*n* = 21)** | **Placebo  (*n* = 15)** | **ANCOVA *F* Value** |
| --- | --- | --- | --- | --- | --- |
| EPA, % |  |  |  |  | 8.05 *** |
| Baseline | 1.09 (0.52) | 1.09 (0.24) | 0.96 (0.41) | 1.10 (0.42) |  |
| Week 16 | 1.85 (0.39) ** | 2.07 (0.77) *** | 1.71 (0.76) * | 1.03 (0.40) |  |
| % change | 69.72 | 89.91 | 78.13 | −6.36 |  |
| DHA, % |  |  |  |  | 2.72 |
| Baseline | 2.74 (1.22) | 2.89 (0.75) | 2.71 (1.10) | 3.03 (0.97) |  |
| Week 16 | 3.98 (0.70) | 3.63 (1.50) | 3.30 (1.63) | 2.71 (1.21) |  |
| % change | 45.26 | 25.61 | 21.77 | −10.56 |  |
| DPA, % |  |  |  |  | 2.00 |
| Baseline | 1.71 (0.81) | 1.95 (0.37) | 1.82 (0.67) | 2.00 (0.46) |  |
| Week 16 | 2.28 (0.28) | 2.29 (0.77) | 2.00 (1.03) | 1.72 (0.70) |  |
| % change | 33.33 | 17.44 | 9.89 | −12.5 |  |
| LCN3 index, % |  |  |  |  | 3.45 * |
| Baseline | 5.53 (2.44) | 5.93 (1.13) | 5.50 (2.06) | 6.08 (1.67) |  |
| Week 16 | 8.11 (1.06) * | 7.98 (2.95) * | 7.01 (3.31) | 5.47 (2.21) |  |
| % change | 46.65 | 34.57 | 27.45 | −10.03 |  |
| AA/EPA, ratio |  |  |  |  | 41.29 *** |
| Baseline | 9.35 (3.25) | 9.72 (2.04) | 10.60 (3.43) | 10.15 (3.22) |  |
| Week 16 | 5.71 (1.36) *** | 4.24 (1.06) *** | 4.54 (1.33) *** | 9.47 (2.84) |  |
| % change | −38.93 | -56.38 | −57.17 | −6.70 |  |
| Total *n*-3, % |  |  |  |  | 3.38 * |

**Table S3**. *Cont.*

| Baseline | 5.72 (2.44) | 6.14 (1.12) | 5.69 (2.08) | 6.29 (1.67) |  |
| --- | --- | --- | --- | --- | --- |
| Week 16 | 8.26 (1.04) * | 8.16 (2.95) | 7.18 (3.29) | 5.67 (2.22) |  |
| %change | 44.41 | 32.90 | 26.19 | -9.86 |  |
| Total *n*-6, % |  |  |  |  | 2.30 |
| Baseline | 21.67 (4.54) | 24.72 (1.35) | 23.63 (5.00) | 24.65 (2.32) |  |
| Week 16 | 22.57 (1.51) | 20.93 (4.90) | 20.41 (5.40) | 24.06 (4.84) |  |
| % change | 4.15 | −15.33 | −13.63 | −2.39 |  |
| *n*-3/*n*-6, ratio |  |  |  |  | 9.19 *** |
| Baseline | 0.25 (0.09) | 0.25 (0.05) | 0.23 (0.07) | 0.25 (0.06) |  |
| Week 16 | 0.37 (0.05) *** | 0.38 (0.10) *** | 0.34 (0.11) ** | 0.23 (0.07) |  |
| % change | 48.00 | 52.00 | 47.83 | −8.00 |  |

Note: EPA = [Eicosapentaenoic](http://en.wikipedia.org/wiki/Eicosapentaenoic_acid) Acid, DHA = [Docosahexaenoic Acid](http://en.wikipedia.org/wiki/Docosahexaenoic_acid), LCN3 = Long Chain Omega-3,
AA = Arachidonic Acid. ANOVA results are for univariate analysis of variance comparing blood values at end-point, by treatment allocation, whilst controlling for baseline values. Stars display results of simple planned contrasts using the placebo as the reference group. * *p* < 0.05, ** *p* < 0.01, *** *p* < 0.001.
